# EFFECTS OF KINESIO TAPING IN PATIENTS UNDERGOING KNEE OR HIP ARTHROPLASTY: A SYSTEMATIC REVIEW AND META-ANALYSIS OF RANDOMIZED CONTROLLED TRIALS

**DOI:** 10.2340/jrm.v57.40784

**Published:** 2025-02-05

**Authors:** Ting MEI, Yuli SHUAI, Dandong WU, Heping YU

**Affiliations:** Department of Rehabilitation, The First Affiliated Hospital of Chongqing Medical University, Chongqing, China

**Keywords:** kinesio taping, meta-analysis, oedema, pain, range of motion, systematic review, total hip arthroplasty, total knee arthroplasty

## Abstract

**Objective:**

To assess the effects of kinesio taping on pain, oedema, range of motion, and joint function following knee or hip arthroplasty.

**Methods:**

Eight databases were searched up to 9 January 2024. Patients undergoing rehabilitation after knee or hip arthroplasty were included. The intervention group received kinesio taping with postoperative rehabilitation, while the control group received postoperative rehabilitation alone. Outcomes for knee arthroplasty patients, included pain, oedema, range of motion, and the Hospital for Special Surgery knee score. For hip arthroplasty, the outcome focused on pain.

**Results:**

Eleven randomized controlled trials involving 774 participants met the inclusion criteria. In knee arthroplasty patients, kinesio taping significantly reduced pain (standardized mean difference [SMD] = –0.53, 95% CI –0.91 to –0.14, *p* = 0.007), and relieved thigh (SMD = –0.38, 95% CI –0.65 to –0.12, *p* = 0.005) and ankle circumferences (SMD = –0.53, 95% CI –0.95 to –0.12, *p* = 0.01). It improved the total range of motion (SMD = 1.26, 95% CI 0.93 to 1.60, *p* < 0.00001) and Hospital for Special Surgery knee score (SMD = 2.17, 95% CI 1.70 to 2.65, *p* < 0.00001). No significant pain intensity reduction was observed in hip arthroplasty patients (*p* = 0.25).

**Conclusion:**

Kinesio taping combined with postoperative rehabilitation effectively reduces oedema and pain, and improves joint function in knee arthroplasty patients, but does not alleviate pain in patients following hip arthroplasty.

## LAY ABSTRACT

Osteoarthritis has become a leading cause of disability worldwide. Advanced stages of osteoarthritis can often be treated effectively with joint arthroplasty. However, after surgery, swelling and pain can significantly hinder joint mobility, leading to stiffness and negatively impacting recovery and quality of life. Kinesio taping, a non-invasive therapeutic technique, is believed to enhance blood circulation and reduce oedema. It is widely employed in the rehabilitation of patients with musculoskeletal conditions, but its effectiveness in reducing pain and swelling and improving knee function remains debated. This systematic review analysed 11 studies involving 774 individuals who underwent total joint arthroplasty. Nine RCTs focused on patients with knee arthroplasty, while 2 studies included patients with hip arthroplasty. Our analysis shows the positive effects of kinesio taping in alleviating postoperative pain, reducing swelling in the lower limbs, and enhancing joint function in patients with knee arthroplasty, but shows no benefits on pain relief in patients after hip arthroplasty.

With the rising population of older adults and individuals with obesity, osteoarthritis (OA) has become a leading cause of disability worldwide (1), particularly in the hip and knee joints (2). Arthroplasty, including total knee arthroplasty (TKA) and hip arthroplasty (THA), is the preferred treatment for advanced OA to alleviate pain and restore function (3). However, postoperative recovery is often hindered by pain (4), swelling (5), and muscle strength loss (6), which restrict movement. Consequently, many patients undergoing TKA or THA report dissatisfaction with their functional recovery (7).

Kinesio taping (KT), a non-invasive therapeutic technique developed by Dr Kenzo Kase in 1973, is designed to enhance blood circulation, reduce oedema, and support muscle function (8). With elasticity mimicking that of human skin, KT facilitates improved range of motion (ROM) and provides a heightened sense of comfort. It is widely used in the rehabilitation of musculoskeletal conditions (9), such as neck or low back pain, rheumatic diseases, and postoperative recovery (10, 11). Although many studies have explored the application of KT in patients after TKA or THA, its effectiveness in reducing pain and swelling and improving knee function remains uncertain, with existing evidence yielding conflicting conclusions (12–14).

To address this gap, we conducted a meta-analysis of randomized controlled trials (RCTs) to evaluate the effects of KT on pain intensity, oedema, ROM, and physical function in patients recovering from TKA or THA.

## METHODS

The systematic review and meta-analysis were reported in accordance with PRISMA guidelines (15) and were registered in advance on PROSPERO (registration number CRD42024504113).

### Search strategy

A systematic search was conducted in PubMed, Cochrane Library, Embase, Web of Science, CBM, China Science and Technology Journal Database (VIP), Wanfang Data, and China National Knowledge Internet (CNKI) to identify relevant studies published in English or Chinese from inception to 9 January 2024. MeSH terms and keywords, including “Kinesio Taping”, “athlete tape”, “knee replacement”, “knee arthroplasty”, “hip replacement” and “hip arthroplasty”, were employed. Detailed retrieval strategies are provided in Appendix S1. Additionally, manual searches were performed to identify further articles potentially missed in the electronic databases.

### Inclusion criteria

Eligible studies were considered if they met the PICOS criteria as follows:

*Participants* (P): Patients undergoing rehabilitation after THA or TKA;

*Interventions* (I): Rehabilitative approaches involving KT administered in combination with other rehabilitation treatments;

*Comparisons* (C): Control group receiving the same postoperative rehabilitation without KT;

*Outcomes* (O): For THA, the outcome focused on a visual analogue scale (VAS). For TKA, outcomes included VAS, numeric rating scale (NRS), knee oedema (knee circumference), ROM, and the Hospital for Special Surgery (HSS) knee score. The HSS (16), which was proposed by the American Hospital in 1976, consists of the following 6 parts: pain, function, range of motion, muscle strength, knee flexion deformity, and stability.

*Study design* (S): Only RCTs were included.

### Exclusion criteria

The exclusion criteria were as follows: (*i*) non-RCTs; (*ii*) reviews, systematic reviews, meta-analyses, letters, clinical registration trials, conference abstracts, or duplicate publications; and (*iii*) incomplete data or original data that could not be obtained.

### Risk of bias and quality assessment

The risk of bias was assessed independently by 2 reviewers (MT and WDD) following the guidelines outlined in the *Cochrane Handbook for Systematic Reviews of Interventions* (17). In cases of disagreement, a third author (YHP) was consulted to reach a consensus. For RCTs, the Cochrane Risk of Bias tool version 2 (RoB 2) was utilized (18). The assessment evaluated the following domains: bias arising from the randomization process, bias due to deviations from intended interventions, bias resulting from missing outcome data, bias in outcome measurement, and bias in the selection of the reported results.

### Data extraction

Data extraction was performed independently by 2 reviewers (MT and WDD) using a customized Microsoft Excel sheet (Microsoft Corp, Redmond, WA, USA). Discrepancies in extracted data were resolved with the involvement of a third auditor (YHP). The extracted data included the following: first author, publication year, country, sample size, average age of control and experimental patients, intervention details for each group, and the follow-up period. Outcome measures such as VAS, NRS, ROM, HSS, and lower-leg circumference were assessed at the end of the intervention and last follow-up.

### Statistical analysis

Review Manager 5.4.1 (https://test-training.cochrane.org/online-learning/core-software-cochrane-reviews/review-manager-revman/download-revman-5) and STATA 16.0 (StataCorp LLC, College Station, TX, USA) were used for all statistical analyses. Standardized mean differences (SMDs) with 95% confidence intervals (CIs) for continuous outcomes were pooled to compare the experimental and control groups in terms of changes in outcome indicators. The I^2^ statistic and Q test (χ^2^) were employed to quantify heterogeneity among the included studies, with a significance level of *p* ≤ 0.05 (19). Random-effect models were used when I^2^ > 50%; otherwise, fixed-effect models were applied. Subgroup analyses were conducted based on intervention time to identify potential sources of heterogeneity. A sensitivity analysis was performed to evaluate the influence of outliers and the robustness of the results by sequentially removing each study. Publication bias was assessed qualitatively using funnel plots and quantitatively using Egger’s test. If the funnel plot was generally symmetric, and Egger’s test indicated *p* > 0.05, publication bias was considered insignificant. When publication bias was significant, the Trim and Fill method was applied for further analysis.

## RESULTS

### Study selection

**Fig. 1:**
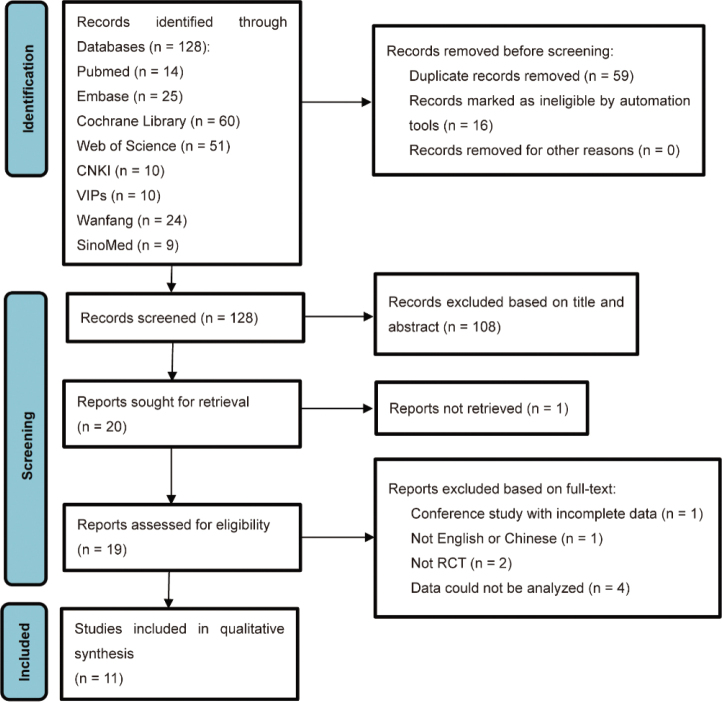
PRISMA flow diagram for search strategy and study selection.

The detailed literature screening process is illustrated in Fig. 1. A total of 203 articles were retrieved, and 128 remained after removing 75 duplicate citations. After screening titles and abstracts, 108 studies were excluded, and 1 study was removed because the full text could not be accessed. Nineteen studies were considered potentially eligible following this process. Eight articles were excluded for the following reasons: 2 studies were not randomized, one study had incomplete data, 1 study was not written in English or Chinese, and data from 4 RCTs could not be analysed. Ultimately, 11 RCTs were included in the systematic review and meta-analysis following a full-text review.

### Study and patient characteristics

Detailed characteristics of the 11 eligible studies are presented in Table I. These studies, involving 774 participants from 5 countries, were analysed, with 382 participants in the KT plus exercise group. The sample size ranged from 27 to 125 participants, and the age range of the patients was 19–87 years. The interventions lasted from 3 days to 3 months. Nine RCTs focused on patients with TKA (12, 13, 20–26), while 2 studies included patients with THA (27, 28). Each article detailed the application, frequency, and duration of KT in the experimental group and exercise regimens in the control group. The application period of KT varied from 14 to 30 days, with a frequency ranging from daily to every 4 days.

**Table I:** Characteristics of the included studies

Study/year	Sample size (*n*)	Mean age (year)	BMI	KT group	Control group	Outcomes	Follow-up time	Main findings
Guney-Deniz et al.,2022, Turkey	K:12C:15	K:66.1 ± 3.50 C:65.4 ± 3.90	K:28.30 ± 2.10 C:29.10 ± 2.70	KTs (2–3 fan-shaped, once for 3 days, POD2-4) + same interventions in control group	POD1-4: cryotherapy + CPM + exercises + medical therapy	Circumference, AROM, VAS	D2, D3, D4, W2, W6 after TKA	Significant difference in VAS and circumference, no significant difference in other outcomes
Cakmak et al.,2023, Turkey	K:62C:63S:64	K:66.11 ± 6.16 C:64.00 ± 8.45S:64.73 ± 7.87	K:30.23 ± 4.41 C:31.14 ± 4.32S:30.75 ± 4.79	KT group: KTs (2 fan-shaped, first time for POD1-3, second time for POD3-7) + same interventions in control group;Shame group: adhesive tape without tension in application	cryotherapy + exercises + CPM + medical therapy	PROM, circumference, VAS	D1, D3, D10 after TKA	Significant difference in circumference, no significant difference in other outcomes
Jarecki et al.,2021, Poland	K:23C:22	K:65.95 ± 7.43 C:66.90 ± 7.44	K:30.60 ± 4.91 C:30.41 ± 6.00	KTs (4 fan-shaped, each KT for 5 days, POD3-8) + same interventions in control group	POD1-8: cryotherapy + CPM + exercises + medical therapy	Knee flexion, VAS	D3, D8 after TKA	Significant difference in circumference, no significant difference in other outcomes
Tornatore et al.,2020, Italy	K:33C:33	K:69.85 ± 5.85 C:71.30 ± 7.09	K:29.14 ± 3.10 C:28.96 ± 3.97	KTs (4 fan-shaped, each KT for 2 days, POD2-6) + same interventions in control group	exercises + MLD + medical therapy	Knee flexion, NRS, circumference	D4, D6 after TKA	Significant difference in NRS and circumference, no significant difference in other outcomes
Donec et al.,2014, Lithuania	K:40C:49	K:66.6 ± 10.5 C:68.1 ± 7.8	K:31.70 ± 5.80 C:33.70 ± 6.10	KTs (2–3 fan-shaped, in POW1–2; 2–3 fan-shaped + Y-strips + I-strip, in POW3–4, each KT for 6 days)+ same interventions in control group	POD1-28: physical therapy + exercises	Circumference, AROM, NRS	D2, D8, D16, D24, D28 after TKA	Significant difference in NRS, extension, and circumference, no significant difference in flexion
Wang,2023, China	K:46C:46	K:47.26 ± 8.21 C:48.21 ± 8.09	ND	KTs (2 fan-shaped, each KT for 2 days, POD1-30)+ same interventions in control group	POD1-30: exercises	VAS, HSS, difference in circumference	D3, D7, D14, D30 after TKA	Significant difference in all outcomes
Xue,2021, China	K:63C:64	ND	ND	KT (4–6 fan-shaped, once/day, each KT for 1 day, POD3–POD14; O-shaped, each KT for 1 day, POD15-POD30)+ same interventions in control group	POD3-POM1: cryotherapy + exercises	ROM, circumference, VAS, HSS	D3, D14, D30 after TKA	Significant difference in all outcomes
Zhu,2011, China	K:17C:16	K:66.30 ± 6.40 C:68.20 ± 5.50	K:26.50 ± 3.10 C:27.00 ± 3.20	KT (Y-shaped + X-shaped, once/day, each KT for 1 day, POD1-4)+ same interventions in control group	POD1-4: exercises	Circumference, AROM, VAS	D1, D2, D3, D4 after TKA	Significant difference in all outcomes
Hu,2023, China	K:20C:20	K:69.42 ± 2.57 C:69.13 ± 2.51	ND	KT (2 fan-shaped + X-shaped + I-shaped + Y-shaped, once/day, each KT for 2–3 days, 6 times/week, POD1-POM3)+ same interventions in control group	POD1-POM3: exercises + CPM + physical therapy	Circumference, VAS, ROM, HSS	M3 after TKA	Significant difference in all outcomes
Yong,2021, China	K:46C:45	K:71.04 ± 5.02 C:72.24 ± 4.42	ND	KTs (I-shaped and Lantern-shaped, each KT for 2 days, POD1-7)+ same interventions in control group	POD1-7: exercises	ROM, VAS	D7 after THA	Significant difference in all outcomes
Wang,2021, China	K:20C:19	K:64.60 ± 13.1 C:65.70 ± 15.1	ND	KT (6–8 fan-shaped + X-shaped, once/day, each KT for 1 day, POD1-5) + same interventions in control group	POD1-5: cryotherapy + exercises + MLD	VAS, circumference	D1, D3, D5 after THA	Significant difference in all outcomes

TKA: total knee arthroplasty; THA: total hip arthroplasty; MLD: manual lymphatic drainage; CPM: continuous passive motion; VAS: Visual Analogue Scale; NRS: Numeric Rating Scale; HSS: Hospital for Special Surgery knee score; EDF: epidermis, dermis, fascia; K/ KT(s): kinesio tape; C: comparison group; S: shame tap group; AROM: active range of motion; PROM: passive range of motion; D: day; W: week; M: month; POD: postoperative days; POW: postoperative weeks; POM: postoperative months; ND: no details.

### Risk of bias and quality assessment

**Fig. 2:**
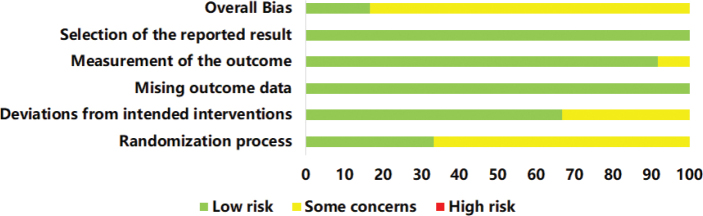
Risk of bias graph of included studies.

**Fig. 3:**
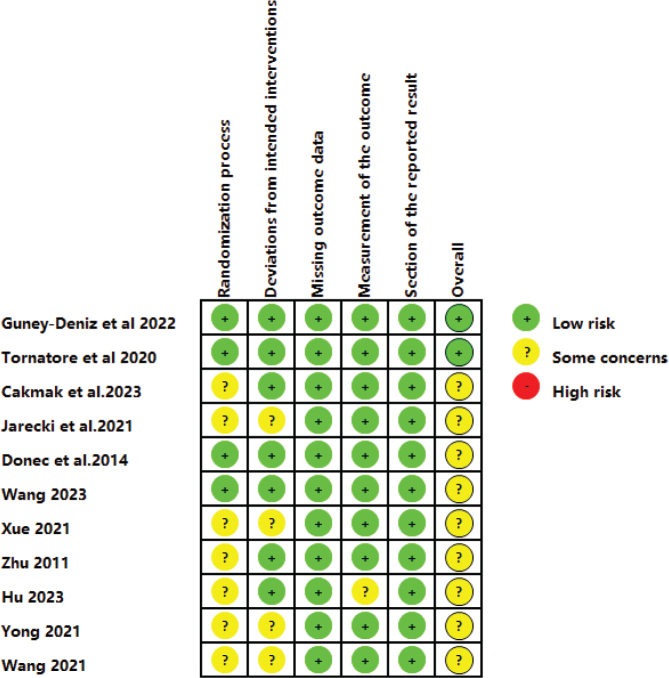
Risk of bias summary of included studies.

A summary of the risk of bias is presented in Figs 2 and 3, illustrating the risk of bias for each included study and for each domain. Green areas indicate a low risk of bias, yellow areas indicate an unclear risk of bias, and red areas represent a high risk of bias. Overall, 2 studies (13, 20) were judged to have a “low risk” of bias, while 9 studies (12, 21–28) were rated as having “some concern”. All included studies were randomized, and provided detailed descriptions of their randomization methods, except 1 study (12), which did not clarify the randomization process. In 4 studies, concerns arose about deviations from the planned interventions due to the lack of blinding of patients and investigators. In summary, the methodological quality of the included studies was generally low to moderate.

### Outcome and analysis

**Fig. 4:**
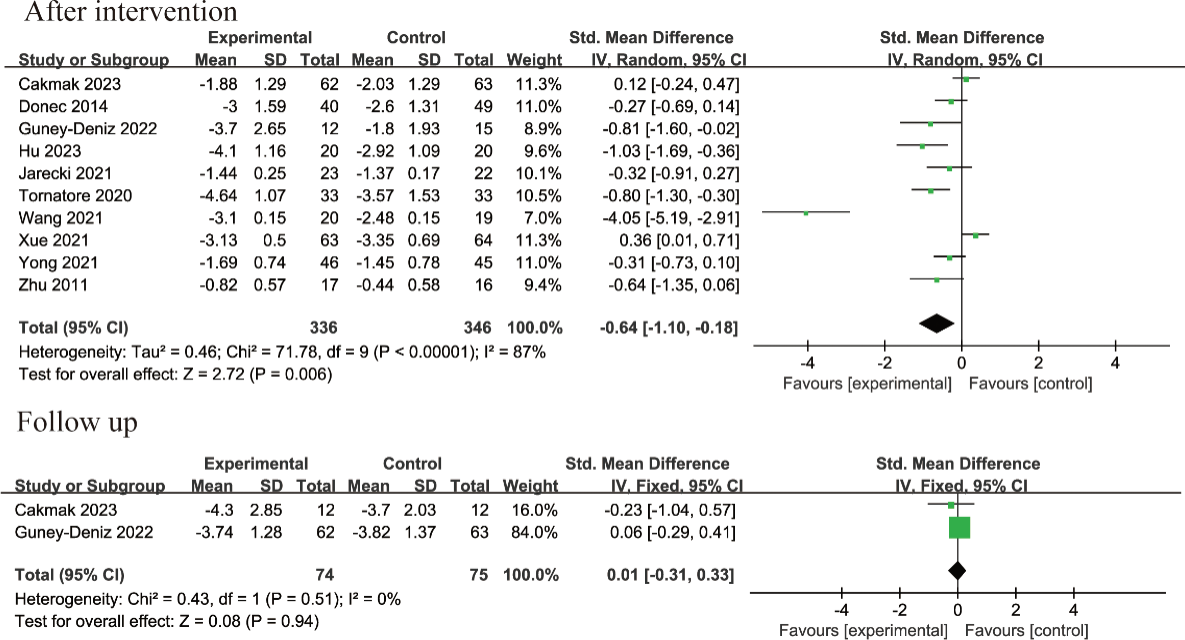
Forest plot of pain intensity in patients with total hip or knee arthroplasty (THA or TKA).

*Pain intensity after surgery:* Postoperative pain was measured using the NRS in 2 studies and the VAS in 8 RCTs. Ten studies (12, 13, 20–24, 26–28) involving 682 patients reported pain intensity after the intervention, while 2 RCTs with 149 patients recorded pain intensity during the follow-up period. A random-effect model was adopted for post-intervention data due to heterogeneity between studies (I^2^ = 87%, *p* < 0.00001). For follow-up data, a fixed-effect model was applied as no discernible heterogeneity was detected (I^2^ = 0%, *p* = 0.51). The results indicated that KT improved pain intensity after the intervention compared with that in non-KT groups (SMD = –0.64, 95% CI –1.10 to –0.18, *p* = 0.006), but no significant difference was observed during the follow-up period (SMD = 0.01, 95% CI –0.31 to 0.33, *p* = 0.94), as shown in Fig. 4.

**Fig. 5:**
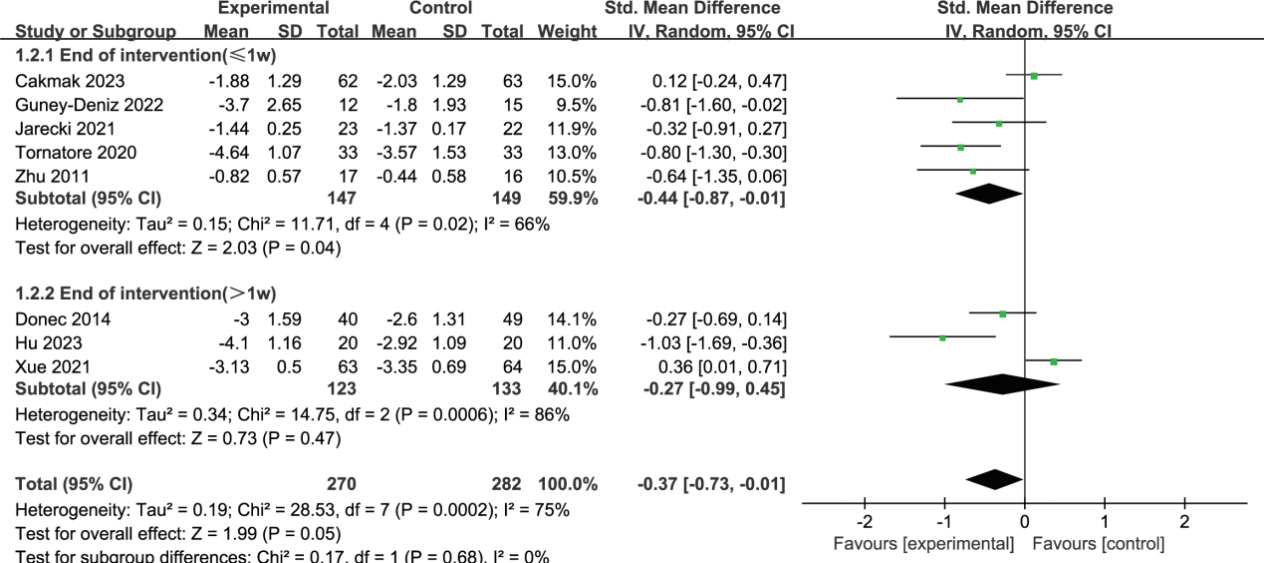
Forest plot of subgroup analysis of pain intensity based on intervention time in patients with TKA.

For RCTs focusing on TKA, 5 studies (12, 13, 20, 22, 26) implemented interventions lasting less than 1 week, while 3 studies (21, 23, 24) had interventions lasting more than 1 week. Subgroup analysis based on intervention time revealed that KT combined with other rehabilitation methods reduced pain more effectively than did rehabilitation without KT when the intervention lasted less than 1 week (SMD = –0.44, 95% CI –0.87 to –0.01, *p* = 0.04). However, no significant difference in pain relief was observed between groups when the intervention lasted more than 1 week (SMD = –0.27, 95% CI –0.99 to 0.45, *p* = 0.47), as shown in Fig. 5.

**Fig. 6:**
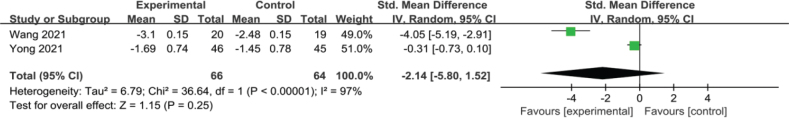
Forest plot of pain intensity after intervention in patients with THA.

For RCTs focusing on THA (27, 28), the intervention times ranged from 5 to 7 days postoperatively. High heterogeneity was observed among the included studies (I^2^ = 97%, *p* < 0.00001), necessitating the use of a random-effect model. Pooled data showed no significant difference between the KT and non-KT groups (SMD = –2.14, 95% CI –5.80 to 1.52, *p* = 0.25), as shown in Fig. 6.

**Fig. 7:**
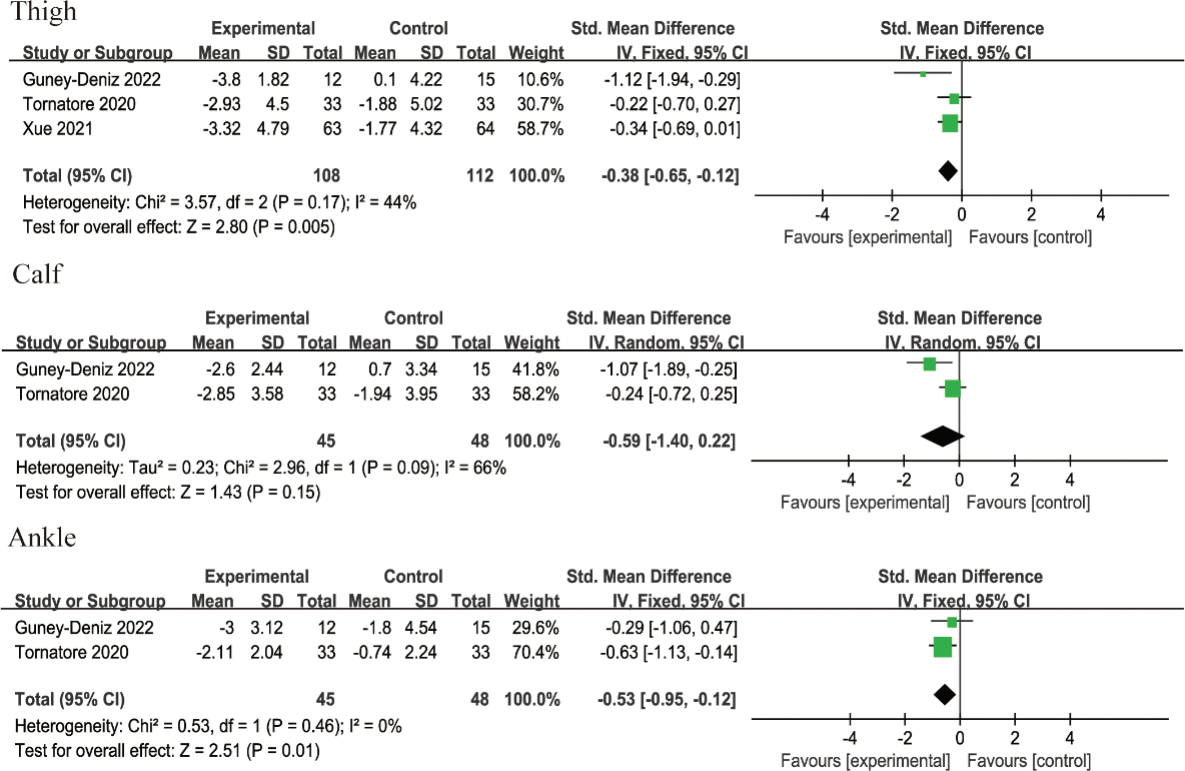
Forest plot of knee oedema after intervention in patients with TKA.

*Knee oedema:* The knee oedema measurements included thigh, calf, and ankle circumferences. Three RCTs reported lower-limb lymphoedema after KT application, involving 220 patients after TKA. The intervention periods were PODs (postoperative days) 2–4 (20), PODs 2–6 (13), and 1 month after surgery (24). Three RCTs (13, 20, 24) provided data on thigh circumferences, while 2 RCTs (13, 20) provided data on calf and ankle circumferences. Low heterogeneity was observed in thigh circumferences (I^2^ = 44%, *p* = 0.17) and ankle circumferences (I^2^ = 0%, *p* = 0.46), and these were analysed using a fixed-effect model. High heterogeneity was identified in calf circumferences (I^2^ = 66%, *p* = 0.09), warranting the use of a random-effect model. The results showed that, compared with non-KT groups, KT significantly reduced thigh circumferences (SMD = –0.38, 95% CI –0.65 to –0.12, *p* = 0.005) and ankle circumferences (SMD = –0.53, 95% CI –0.95 to –0.12, *p* = 0.01). However, no significant benefit was observed for calf circumferences (SMD = –0.59, 95% CI –1.40 to 0.22, *p* = 0.15), as shown in Fig. 7.

**Fig. 8:**
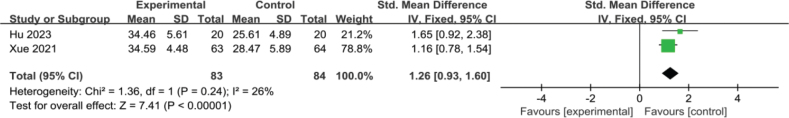
Forest plot of ROM of the knee joint after intervention in patients with TKA.

*ROM of the knee joint:* Two studies reported the ROM after intervention, involving 167 patients after TKA. The intervention periods were 1 month (24) and 3 months (23). Low heterogeneity was observed among the included studies (I^2^ = 26%, *p* = 0.24) and a fixed-effect model was applied. The meta-analysis revealed that KT significantly improved knee ROM (SMD = 1.26, 95% CI 0.93 to 1.60, *p* < 0.00001), as shown in Fig. 8.

**Fig. 9:**
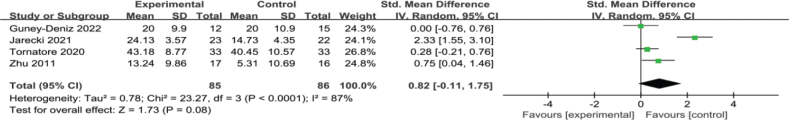
Forest plot of knee flexion after intervention in patients with TKA.

*Flexion ROM:* Four RCTs (12, 13, 20, 26), studies involving 171 patients, provided data on flexion ROM after intervention. All interventions were conducted within 1 week postoperatively. High heterogeneity was observed among the studies (I^2^ = 87%, *p* < 0.0001), necessitating the use of a random-effect model. The overall estimate indicated no statistically significant difference between the KT and control groups in patients after TKA (SMD = 0.82, 95% CI = 0.11 to 1.75, *p* = 0.08), as shown in Fig. 9.

**Fig. 10:**
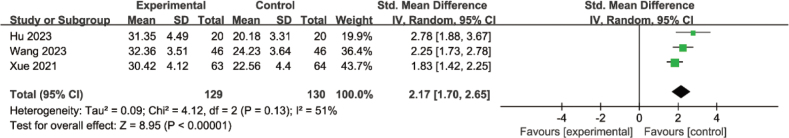
Forest plot of knee function score (HSS) in patients with TKA.

*Knee function score:* Postoperative knee function was measured by the HSS. Three RCTs (23–25) involving 256 patients reported postoperative knee function scores at the end of the intervention. The intervention durations varied from 1 month (24, 25) to 3 months (23). As shown in Fig. 10, the HSS score in the experimental group was significantly higher than that in the control group in patients after TKA (SMD = 2.17, 95% CI 1.70 to 2.65, *p*<0.00001).

### Sensitivity analysis

**Fig. 11:**
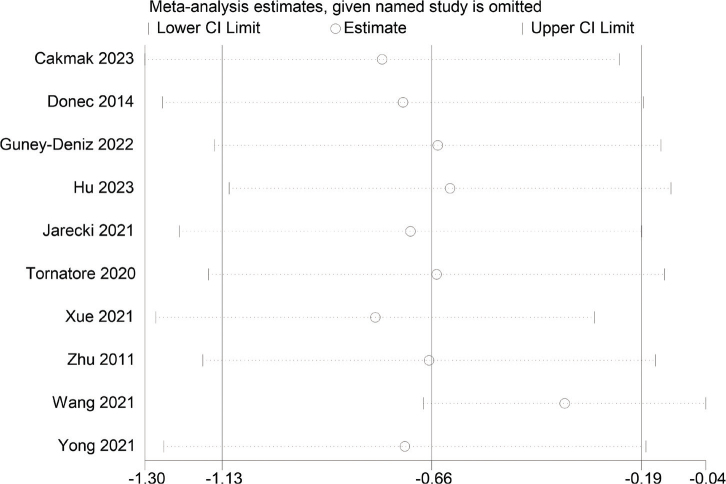
Sensitivity analysis of pain intensity after intervention.

A sensitivity analysis of the meta-analysis was conducted, indicating no considerable variation of the pooled results after the exclusion of each RCT one after another. It indicated that the results of meta-analysis were relatively robust (Fig. 11).

### Publication bias

**Fig. 12:**
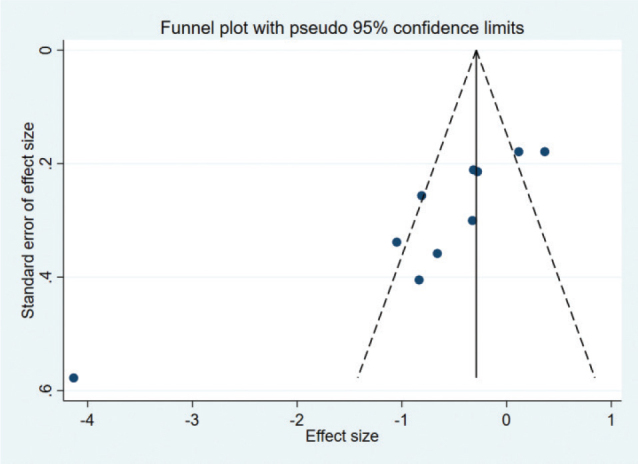
Funnel plot of pain intensity scores.

**Fig. 13:**
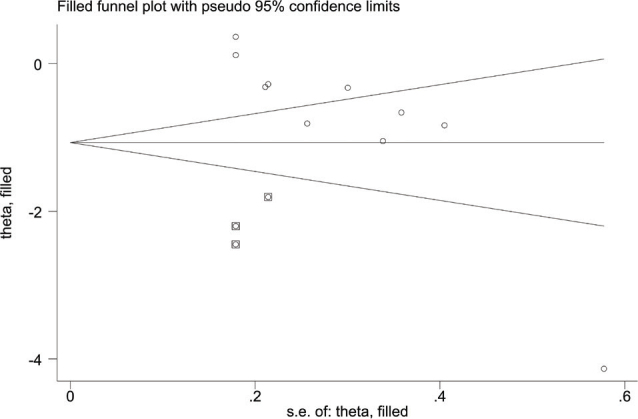
Filled funnel plot of pain intensity scores.

For pain intensity, the funnel plot was not symmetric (Fig. 12), and the *p*-value for Egger’s test was 0.001 (< 0.05), indicating the presence of publication bias due to asymmetry. The asymmetrical funnel plot was corrected using the Trim and Fill method. After 5 iterations, 3 articles were imputed to adjust the result. The final result (SMD = –1.068, 95% CI –1.689 to –0.447, *p* = 0.001; Fig. 13) was consistent with the direction of the original result.

## DISCUSSION

KT has been widely adopted in the rehabilitation of musculoskeletal disorders to reduce oedema, alleviate pain, and improve joint function. KT exerts its effects by lifting the skin and expanding the subcutaneous space, facilitating enhanced blood and lymphatic circulation, which in turn attenuates oedema and reduces pain (29, 30). Additionally, KT enhances proprioception and alleviates pain by stimulating cutaneous mechanoreceptors (31, 32).

The present meta-analysis of RCTs demonstrated that KT was associated with significant improvements in pain, swelling, ROM, and knee function following knee arthroplasty, but no significant improvement in the pain intensity in hip replacement patients. Pain is a common complication after TKA, and uncontrolled pain can delay the recovery process and lead to chronic pain (33). Thus, effective pain management is critical. This meta-analysis revealed that in patients with TKA, KT combined with postoperative therapy significantly reduced pain when the intervention period was less than 1 week. However, no significant reduction in pain was observed when the intervention period exceeded 1 week or during the follow-up period. In other words, KT was effective for short-term pain relief in the immediate postoperative period but showed no significant long-term benefits, which is consistent with previous findings (34–37). KT lifts the skin, which increases blood circulation and lymphatic drainage to reduce swelling, and also to reduce pressure on subcutaneous nociceptors, thereby alleviating pain (29, 30). Acute swelling is believed to be closely associated with pain in the early postoperative stage. As KT significantly alleviates the acute swelling, it may consequently relieve pain. During our meta-analysis on pain intensity, we identified a significant outlier in Wang’s (2021) study that substantially influenced the results. Several factors may explain this discrepancy. First, the interventions in Wang’s study included manual lymphatic drainage, which differs from the interventions in other included studies. Second, Wang’s research focused on hip arthroplasty, whereas most other studies focused on knee arthroplasty. To address this, we conducted a subgroup analysis specifically examining postoperative pain following hip arthroplasty. The analysis revealed no significant improvement in pain for patients with THA. Only 2 studies reported on this outcome, and differences in KT application methods may have contributed to the high heterogeneity. Additionally, we noted that the included studies lacked detailed information regarding analgesic medications. Variations in medication types and administration protocols could have further influenced the pain intensity outcomes. Future research should aim to address these limitations by standardizing intervention protocols and providing comprehensive details on concomitant treatments.

Lower-extremity swelling contributes significantly to dissatisfaction with functional recovery following TKA. Untreated oedema may exacerbate pain, impair mobility, and increase the risk of infection (38). The pooled data demonstrated that KT effectively alleviated oedema. KT has been shown to be a beneficial method for reducing lymphoedema by accelerating lymphatic and venous microcirculation (39). Previous studies have also highlighted the positive effects of KT on oedema reduction in patients undergoing anterior cruciate ligament reconstruction (40) and in those with breast cancer-related lymphoedema (41). Combining our data with prior evidence, we maintain a positive outlook regarding the efficacy of KT in reducing oedema. However, it is important to note that only 3 studies measured this outcome, and differences in circumference measurement methods may have contributed to the observed heterogeneity. Future research should employ more precise assessment techniques for evaluating oedema severity, such as volumetric measurements or ultrasound-assisted evaluations, to enhance the reliability and comparability of findings.

Swelling and pain following TKA can significantly impede knee joint mobility and lead to joint stiffness, adversely i mpacting patients’ quality of life. Previous evidence suggests that KT application improves ROM, particularly in patients with OA (42, 43).

However, the current study presents inconsistent findings. While KT was shown to significantly impact knee ROM, no significant difference in joint flexion was observed between the groups. Our data indicate that KT may influence knee extension rather than flexion in patients undergoing TKA. We propose several potential reasons for this observation. In the early postoperative stage, swelling and pain can cause quadriceps muscle inhibition, leading to reduced quadriceps strength. Studies report an average loss of 60–83% of knee extension strength in the operated leg during this period (6, 44). By alleviating postoperative pain and swelling, KT may help restore quadriceps strength, potentially contributing to improved knee extension.

To evaluate the overall effectiveness of KT in patients who underwent TKA, we explored the knee function following KT application. Our findings showed an improvement in HSS scores in those patients treated with KT. As the HSS scale encompasses multiple factors, including ROM, pain, muscle strength, and other parameters, the KT-associated improvement in HSS scores may result from combined effects. However, variations in the timing of evaluations across studies may contribute to the observed heterogeneity.

To our knowledge, this is the most recent review and meta-analysis comparing the efficacy of KT plus exercise therapy with that of exercise alone in patients undergoing TKA or THA. However, some limitations must be acknowledged: (1) although this study adhered to PRISMA guidelines, the risk of selection bias may persist. For instance, only studies published in Chinese and English were included, potentially excluding relevant studies in other languages; (2) the number of included studies was limited, with only 11 RCTs with sample sizes ranging from 27 to 125 participants. Certain outcomes, such as pain intensity following THA, were analysed based on relatively small sample sizes. These results should be interpreted with caution; (3) most studies did not include a placebo intervention, making it challenging to rule out the potential influence of placebo effects; (4) heterogeneity was high due to the diverse types of KT techniques applied in the included studies; (5) variations in rehabilitation training content and medication usage across studies may have affected the accuracy and generalizability of the results; (6) some measurement methods for outcomes, such as manual circumference assessments, are prone to human error. Employing more objective and standardized measurement techniques, such as volumetric analysis or ultrasound-based evaluations, is recommended to improve result reliability. To overcome these limitations, future research should include larger sample sizes and employ more rigorous, high-quality RCTs that focus on KT applications and standardized methodologies.

In conclusion, this review and meta-analysis shows the positive effects of KT on postoperative pain intensity, lower-limb swelling, and joint function in patients undergoing TKA, but shows no benefits on pain relief in patients after THA. Future research should prioritize large-scale, high-quality RCTs with standardized outcome indicators and rigorous methodologies.
